# Hypothyroidism Affects Olfactory Evoked Potentials

**DOI:** 10.1155/2016/9583495

**Published:** 2016-08-30

**Authors:** Teodor Świdziński, Kamila Linkowska-Świdzińska, Hanna Czerniejewska-Wolska, Bożena Wiskirska-Woźnica, Maciej Owecki, Maria Danuta Głowacka, Anna Frankowska, Katarzyna Łącka, Mariusz Glapiński, Zofia Maciejewska-Szaniec, Piotr Świdziński

**Affiliations:** ^1^Department of Biophysics, ul. Fredry 10, 61-701 Poznań, Poland; ^2^Department of Conservative Dentistry and Periodontology, University of Medical Sciences, ul. Bukowska 70, 60-567 Poznań, Poland; ^3^Department of Phoniatrics and Audiology, University of Medical Sciences, ul. Przybyszewskiego 49, 60-355 Poznań, Poland; ^4^Department of Endocrinology, Metabolism and Internal Medicine, University of Medical Sciences, ul. Przybyszewskiego 49, 60-355 Poznań, Poland; ^5^Department of Organization and Management in Health Care, University of Medical Sciences, ul. Smoluchowskiego 11, 60-179 Poznań, Poland; ^6^Department of Oral Rehabilitation, Division of Prosthodontics, University of Medical Sciences, ul. Bukowska 70, 60-567 Poznań, Poland

## Abstract

*Background*. Objective electrophysiological methods for investigations of the organ of smell consist in recordings of olfactory cortex responses to specific, time restricted odor stimuli. In hypothyroidism have impaired sense of smell.* Material and Methods*. Two groups: control of 31 healthy subjects and study group of 21 with hypothyroidism. The inclusion criterion for the study group was the TSH range from 3.54 to 110 *μ*IU/mL.* Aim*. Assessment of the latency time of evoked responses from the olfactory nerve N1 and the trigeminal nerve N5 using two smells of mint and anise in hypothyroidism.* Results*. The smell perception in subjective olfactory tests was normal in 85% of the hypothyroid group. Differences were noticed in the objective tests. The detailed intergroup analysis of latency times of recorded cortical responses *P*_N5_ and *P*_N1_ performed by means between the groups of patients with overt clinical hypothyroidism versus subclinical hypothyroidism demonstrated a significant difference (*p* < 0.05) whereas no such differences were found between the control group versus subclinical hypothyroidism group (*p* > 0.05).* Conclusion*. We can conclude that registration of cortex potentials at irritation of olfactory and trigeminal nerves offers possibilities for using this method as an objective indicator of hypothyroidism severity and prognostic process factor.

## 1. Introduction

Hypothyroidism, condition in which the thyroid fails to produce a sufficient amount of hormones, manifests itself mainly in the form of general constitutional symptoms such as fatigue, constipation, increased sensitivity to cold, weight gain, thinning hair, skin dryness, and hoarseness of voice. The symptoms of a nervous system disorder, most frequently in the form of peripheral neuropathy, do not manifest themselves in a way that significantly impairs the patient's ability. Peripheral neuropathy may occur particularly in the course of severe and persistent untreated hypothyroidism. Although the relationship between hypothyroidism and peripheral neuropathy is not entirely understood, it is known that hypothyroidism may cause fluid retention in the tissues and thus exert pressure on peripheral nerves [1–3].

Peripheral neuropathy may, in turn, be one of the causes of olfactory disorders. The studies by Mackay-Sim and Beard conducted on mice indicate that thyroxine is necessary for normal development of the nervous system, including the genesis of new olfactory receptor neurons [4, 5]. Although hypothyroidism disrupts development of the olfactory epithelium, it does not cause however complete atrophy of neurons [6]. Olfactory disorders are most frequently caused by conduction disorders of the sensory stimulus mainly due to upper respiratory tract infections, infections of the nose and sinuses, and injuries or as an idiopathic disorder [7–9]. In our study we excluded this group of patients.

In primary hypothyroidism, disorders of smell and taste turn out to be frequent pathologies [10], which is confirmed also by other researchers who indicate that hypothyroidism significantly influences smell perception attenuating or even suppressing it completely. There is, however, little information considering the relationship between severity of hypothyroidism and intensity of smell disorders [11–13]. It seems evident therefore that a distinction should be drawn between the stages of smell disorders depending on the form of hypothyroidism: mild subclinical versus overt clinical forms. Olfactory reactions that can be disturbed in thyroid diseases are difficult to assess subjectively, without the use of specialist equipment. In view of the above, the diagnostic tests used in olfactory disorders are divided into subjective and objective methods, wherein objective methods (in contrast to subjective tests assessing the thresholds of smell perception) are based on changes in registration of smell cortex evoked potentials, characterized by greater accuracy and repeatability [7, 14–16].

Since the impact of subclinical hypothyroidism on the sense of smell seems to be unclear, and due to the fact that there have been no distinct differences drawn in this respect between overt and subclinical forms of hypothyroidism, the aim of this work was to evaluate the relationship between intensity of hypothyroidism and the state of olfactory reactions measured by objective method.

## 2. Material and Methods

The material comprised 2 groups: the first group consisted of 31 healthy subjects aged 24 to 66 years (mean 51 years) without symptoms of smell disorders displaying a free access to the olfactory region in the nasal cavity in the rhinological test, and the other study group included 21 patients aged 24 to 71 years (mean 54 years) with hypothyroidism. Prior to commencement of the olfactory tests, an informed consent was obtained from each participant to undergo the procedures, and subsequently the subjects received complete ear nose and throat examination to exclude cases of smell disturbances such as nasal and sinus disease, upper respiratory tract infection, head trauma, atrophy of nasal mucosa, and cases with taste disorders.

All the subjects underwent subjective threshold tests of smell perception determined by Ellsberg's olfactory test modified by Pruszewicz and olfactory objective tests recording latency times of responses from cranial nerves I and V using two smells of mint and anise.

The inclusion criterion for the study hypothyroid group was the TSH range from 3.54 to 110 *μ*IU/mL. The subclinical form was determined for 3.51 ≤ TSH ≤ 10 *μ*IU/mL; values above the limit were regarded as representing the overt clinical form. All patients underwent examination by two endocrinologists to confirm the diagnosis and all of them were enrolled into the study at the time of diagnosis. In each patient, signs and symptoms of various degrees of hypothyroidism were found, with a broad range of intensity that increased in line with TSH levels. We observed the following clinical symptoms and signs: weight gain, hair loss, dry skin, decreased libido, easy fatigability, menstrual abnormalities (both oligo- and polymenorrhea and metrorrhagia), peripheral edema, hoarse voice, memory, and concentration problems. Importantly, all the smell examinations shown in this paper were performed prior to the administration of levothyroxine treatment. The reference group consisted of 31 subjects matched for age with the study group for whom the TSH value was normal and ranged from 0.1 to 3.50 *μ*IU/mL. In none of them any signs or symptoms of thyroid disease were found.

Disturbances in perception thresholds of the olfactory test within the subjective scale were evaluated using Ellsberg's olfactory method modified by Pruszewicz [13, 17]. The method is however subjective, depending on individual perception of the patient. Measurements based on recordings of smell cortex potentials are more accurate.

The subjective Ellsberg method modified by Pruszewicz was used primarily for selection of the control group and to obtain preliminary information on the state of the organ of smell in the other participants of the experiment. Thresholds of perception for those with a normal sense of smell determined by Pruszewicz for both oils of mint and anise are 12 mL. It is the volume of the saturated vapors of these oils at room temperature about (22 ± 1)°C administered with a syringe in about 0.5 sec into each nostril separately. Identical quality and the production of these oils according to the manufacturer—The National Chemical Reagents POCh—are guaranteed for 50 years [9, 15]. In the control group the scope of the thresholds of perception for both anise and mint oils ranged in 3–8 mL. The method of registering olfactory cortical response by T. Świdziński was used with similar stimulus lasting for 0.5 s with speed of 10–30 mL/s. This range of applied speed of stimulus provides optimal recording cortical olfactory response. For speeds less than 5 mL/s single questionable entries for the control group were obtained, whereas at speeds greater than 50 mL/sec single false responses from the trigeminal nerve endings during the anise oil examination were registered. It can be suggested that they were caused by nonspecific mechanical or thermal stimulants.

The apparatus for recordings of ERP (evoked reaction potentials) as well as the authors' self-designed device that enables appropriate dispensing of olfactory stimuli were used [15]. The modified self-designed device for objective measurement of cortex reaction potentials evoked by olfactory stimuli, which proves to be a unique investigation, is at present routinely used in our center in diagnostics and clinical evaluation of the organ of smell.

Application of the olfactory stimulus is synchronized with the inspiration phase of the subject in our study. Automatically, using the vacuum sensor that reacts to the onset of each inspiration, the olfactory applicator starts the device for recording the averaged evoked responses.

The ERA 2250 apparatus by Madsen Electronics is used to record evoked responses by means of Beckman electrodes placed to the forehead and bilaterally to the nape (or the neck). The technique of summing and averaging responses to a quantitatively identical stimulus was used. The number of repetitions was 10 stimuli, and the response recording time ranged from 0 to 1000 ms. Olfactory stimuli (anise, mint) were used for the tests in the volumes of 5 and 10 cm^3^ ranging within the norms determined by Pruszewicz for the modified Ellsberg's method [13] and additionally 15 cm^3^. Anise oil stimulated endings of the olfactory nerve and mint oil endings of the olfactory and trigeminal nerves. It was possible to differentiate responses to stimuli irritating nerve V endings (potential *P*_N5_ within latency range 200–410 ms) as well as nerve I endings (potential *P*_N1_ within latency range 460–700 ms). The Kruskal-Wallis, Mann-Whitney's significant differences tests, descriptive statistical tests, and Spearman's rank correlation tests were used.

Results of the objective olfactory tests in hypothyroid patients (both forms of hypothyroidism) were compared to the results obtained from healthy subjects from the reference group. Moreover, the effect of increased TSH on the latency of smell cortex potentials *P*_N1_ and *P*_N5_ in the subjects with 2 forms of hypothyroidism was evaluated.

## 3. Results

The groups markedly differed in TSH levels; the patients were divided into groups with different TSH in *μ*IU/mL levels: first, control group interval between minimum 0.01 and maximum 3.01, median 1.23; second group, subclinical form, interval between minimum 3.54 and maximum 10.00, median 5.80; and the third group, clinical overt form, interval between minimum 12.90 and maximum 110.00, median 20.51. The significance of differences test (Kruskal-Wallis) in these groups was *p* ≪ 0.001.

In the subjective olfactory tests performed using Ellsberg's olfactory test method modified by Pruszewicz in the hypothyroid patients, the smell perception thresholds (mint and anise) were normal in 85% cases.

In the remaining 15% of our patients we found abnormal results of subjective tests of smell perception (mint, anise) thresholds, as well as a lack of cortical potentials in electrophysiological registration after olfactory stimulation (5% of cases) or slight cortical responses with very late latencies, over 400 ms (10% of cases).

However, differences were noticed in the objective tests in which evaluation concerned recordings of electric responses to olfactory stimulation of nerves N1 and N5 by means of aromatic smells, mint and anise in groups of healthy subjects and hypothyroidism.

Table 1 shows mean values, medians, ranges, and standard deviations of recorded latency times of olfactory potentials for both study groups and the reference group. As seen there, the mean latencies of cortical responses (*P*_N1_ potential) in the study groups differ significantly in the Kruskal-Wallis test for stimulation with mint oil (*p* < 0.05) and with anise oil (*p* < 0.005).

A similar dependence was found (*p* < 0.05) at cortical recording for nerve V stimulation with mint oil (*P*_N5_ potential). Simultaneously, however, differences between the mean latency values of the studied potentials in the subclinical hypothyroid group are not statistically significant in comparison to the healthy controls (Mann-Whitney's test *p* > 0.05).

**Figure 1:**
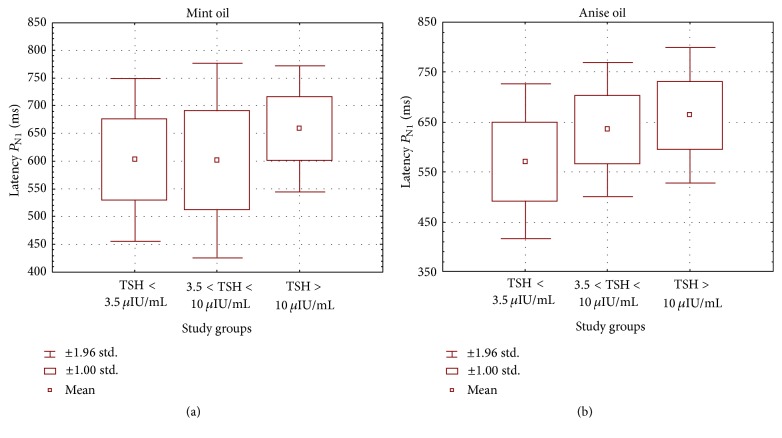
Mean values, ranges, and standard deviations of cortical response latencies in the study groups of hypothyroid patients (subclinical group 3.51 ≤ TSH ≤ 10 *μ*IU/mL and clinical group TSH > 10 *μ*IU/mL) and healthy subjects (TSH < 3.5 *μ*IU/mL) for *P*_N1_ potential at olfactory stimulation with (a) mint and (b) anise.

**Figure 2:**
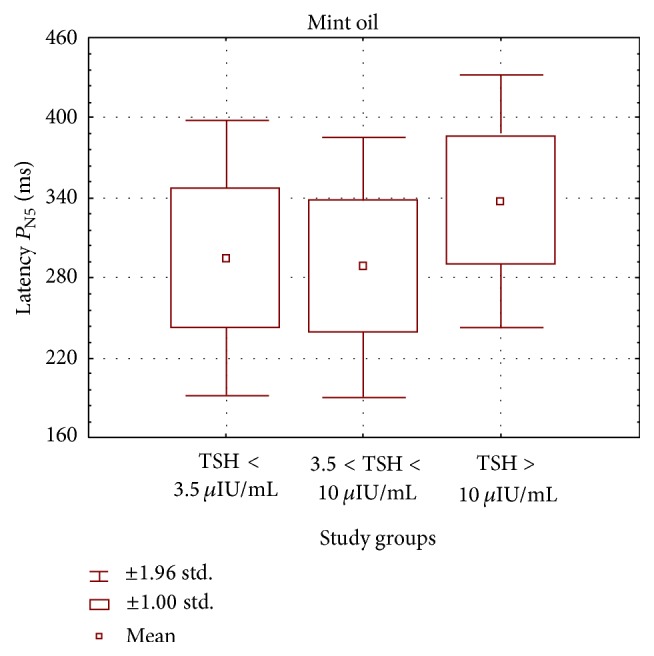
Mean values, ranges, and standard deviations of cortical response latencies in the study groups of hypothyroid patients (subclinical group 3.51 ≤ TSH ≤ 10 *μ*IU/mL and clinical group TSH > 10 *μ*IU/mL) and healthy subjects (TSH < 3.5 *μ*IU/mL) for *P*_N5_ potential at olfactory stimulation with mint.

Graphs in Figures 1 and 2 present a comparison of mean values, ranges, and standard deviations of *P*_N1_ and *P*_N5_ potentials in the subclinical and overt clinical hypothyroid groups as well as in controls.

Analysis of the correlation between TSH values and latencies of smell cortex evoked responses to olfactory and trigeminal nerves stimulation indicated that the greater the TSH value, the longer the latency of the recorded potential.

**Figure 3:**
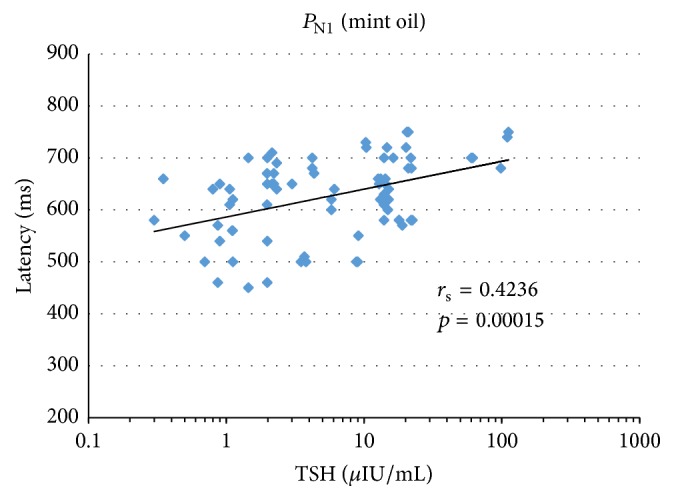
Results of the correlation between TSH and latency of smell cortex evoked potentials *P*_N1_ at mint oil stimulation.

**Figure 4 fig4:**
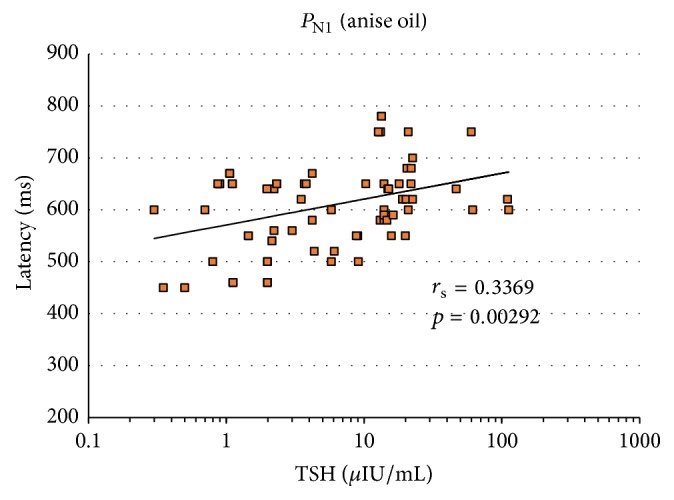
Results of the correlation between TSH and latency of smell cortex evoked potentials *P*_N1_ at anise oil stimulation.

**Figure 5:**
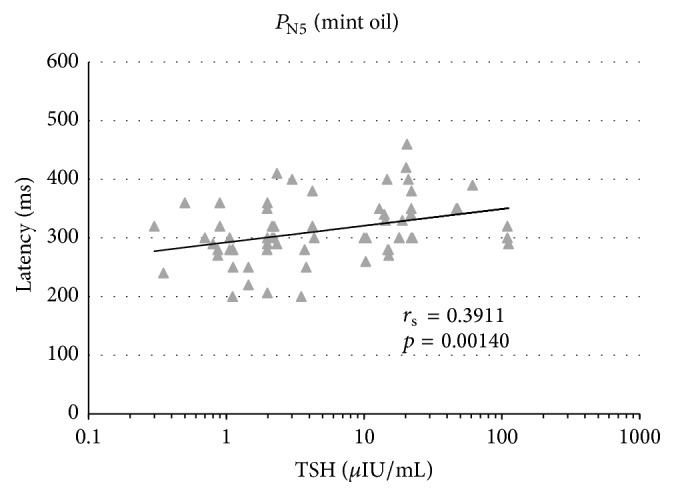
Results of the correlation between TSH and latency of smell cortex evoked potentials *P*_N5_ at mint oil stimulation.

Figures 3 [Fig fig4]–5 show correlations between the parameters (TSH versus latency) under analysis for all the three study groups of subjects. We also presented here the value of the correlation coefficient *r*_s_-Spearman's rank for significance of *p*.

The growing trend (visible in Figures 3 [Fig fig4]–5) between TSH and latencies for the analyzed potentials proves to be a statistically significant relationship for *P*_N1_ at mint and anise stimulation (Figures 3 and 4) as well as for *P*_N5_ at mint stimulation (Figure 5).

Spearman's rank correlation coefficient that served to describe the correlation strength of two measurable features, namely, TSH values and latencies of cortical responses, demonstrated a strong* positive* correlation between increased TSH and prolonged latencies for potential *P*_N1_ at olfactory stimulation with mint and anise (*r*_s_ = 0.42 and 0.34) as well as for potential *P*_N5_ at olfactory stimulation with mint (*r*_s_ = 0.39).

Furthermore, the analysis of latency times of recorded cortical responses *P*_N1_ and *P*_N5_ performed with the Mann-Whitney *U* test between the groups of patients with overt clinical hypothyroidism versus subclinical hypothyroidism demonstrated a significant difference (*p* < 0.05) whereas no such differences were found between the control group versus subclinical hypothyroidism group (*p* > 0.05).

Detailed results are presented in Table 2.

Moreover, it was also observed that above the TSH limit = 30 *μ*lU/mL there were no recorded cortical responses in as many as 70% cases, particularly as regards potential *P*_N5_, despite the fact that, in the subjective tests, normal threshold values of olfactory perception were obtained.

In addition, detailed results of the studies demonstrated also that there were no differences in latency times for *P*_N1_ as well as *P*_N5_ in the hypothyroid group and in the reference group for different values of olfactory stimulations in cm^3^ (5, 10, 15 cm^3^) both at stimulations with anise oil as well as mint oil (Kruskal-Wallis test *p* > 0.05).

It was noted, however, that irrespective of the volume of inhaled stimulus fragrance, rapid olfactory fatigue was observed. Therefore, the number of olfactory stimuli for an averaged response was limited to 10 repetitions in our tests.

## 4. Discussion

Apart from an effect on subjective perception of fragrances, a symptom of olfactory disorders proves to be a change in parameters of objective ERP recording of smell cortex potentials such as latency time or response amplitude. A subjective olfactory perception does not always correlate with objective tests of the central nervous system (CNS) responses [18].

The methods used in clinical practice for objective examination of the olfactory function are based on recording reflex reactions. Such methods are, for example, so-called reflex olfactometry (olfactorhinometry according to Gundziol and Mlynski [7]), changes in cerebral bioelectrical function (EEG olfactometry acc. to Roux and Synek [14]) and recording of smell cortex potentials (as averaged responses to strictly normalized olfactory stimuli in Hummel's method [16]), and the method by Świdziński [15]. The latter was used to test hypothyroidism. The main problem in computer-based olfactometry that consists in averaging cortex potentials is the manner of stimulation [14, 19, 20].

A considerable difficulty seems to be how to ensure repeatable application of olfactory impulses. There have been introduced, however, the so-called impulse olfactometers in which it is possible to control parameters of the stimulus [19, 21, 22], yet another difficulty seems to be elimination of simultaneous irritation of other afferent endings [23].

The following authors were able to overcome this difficulty. Fikentscher, who had used the olfactory method according to Ellsberg, obtained clear cortex responses with latency time 500–1000 ms [24]. Making use of the impulse olfactometer with the electronically amplified olfactory stimulus (designed by Giesen and Mrowiński), Alber et al. (1972) made computerized averaging of 1.5- second electroencephalogram sections in responses to an olfactory stimulus [25]. Then, Herberhold obtained cortex evoked olfactory potentials using 10–20 mL stimuli with 100–200 ms stimulation time. He obtained two separate potentials of 250 ms latency time for responses from the trigeminal nerve and 500 ms for those from the olfactory nerve [8].

Methods of recording the potentials of smell used by us turned out to be accurate, and problems with stimulation of the olfactory organ were overcome. This objective olfactometry method is author's method [15].

The objective test method for mint and anise fragrance stimulation that has been used in our center for several years providing us with wide clinical experience appears to comply with the requirements of an objective investigation. Implementing this method in diseases of the upper respiratory tract as a complementary method, for instance, in injuries of the craniofacial skeleton and surgical operations of the nasopharyngeal tumors, has already become quite common [5–7, 26, 27].

Hence, there have been further attempts to spread the use of this method to disease entities that may be accompanied by peripheral neuropathies even without clear clinical symptoms of olfactory disorders, for example, in neurological diseases such as Alzheimer's, Parkinson's, and Creutzfeldt-Jakob's diseases [28].

As obtained in our study, disturbances of latency response to cortical stimulation on the olfactory nerve and trigeminal probably are related to disturbances of the cortex not receptor. Studies of other authors using more sophisticated methods to stimulate the olfactory structures (such as pyridine and nitrobenzene) affirm opportunity for differentiation of sensory or cortical disorders [10, 29]. This is all more understandable that in the majority of respondents olfactory functions on discrete scents of mint and anise were not disturbed, though we found also in all this cases delayed latencies of cortical potentials *P*_N1_  and  *P*_N5_.

To date, no studies have been undertaken to analyze latencies of olfactory responses recorded from nerves I and V in hypothyroidism in both subclinical and overt forms.

The method designed for receptor stimulation in the olfactory region of the nasal cavity as well as the analysis of latencies of smell cortex evoked potentials allow us to differentiate responses to stimulation of N5 endings (shorter latency) and N1 endings (longer latency) and can become useful in monitoring changes in olfactory disorders caused by hypothyroidism.

There seems to be no immediate link between subjective perception of fragrance and the delay of recorded smell cortex potentials in different forms of hypothyroidism.

In this paper we show for the first time that the greater the TSH values, the longer the latency of smell cortex potentials recorded from both the trigeminal nerve N5 and the olfactory nerve N1.

## 5. Conclusions

The results from analyses of the obtained recordings allow us to conclude that registration of cortex potentials at irritation of olfactory and trigeminal nerves offers possibilities for use in clinical practice as one of the basic methods in objective olfactometry, and its use in hypothyroidism as an objective indicator of hypothyroidism severity may prove helpful in the diagnostic and prognostic processes.

## Figures and Tables

**Table 1:** Mean values, ranges, and standard deviations of recorded latency times of olfactory potentials *P*_N1_ and *P*_N5_ for all the groups of the subjects in stimulation with concentrated vapors of mint and anise oils.

Latency in ms	Hypothyroidism						Reference group (norm)		
	Subclinical			Overt clinical					
	*P*_N1_ mint	*P*_N1_ anise	*P*_N5_ mint	*P*_N1_^*∗*^ mint	*P*_N1_^*∗∗*^ anise	*P*_N5_^*∗*^ mint	*P*_N1_ mint	*P*_N1_ anise	*P*_N5_ mint
Mean	597	588	291	658	667	337	603	571	301
Minimum	500	500	200	570	550	270	450	450	200
Maximum	730	720	380	750	780	460	710	670	410
Standard deviation	88.1	72.6	55.0	58.8	71.8	51.9	77.1	78.9	55.3

^*∗*^ *p* < 0.05, ^*∗∗*^ *p* < 0.005 (Kruskal-Wallis test).

**Table 2:** Difference significance test in latencies of recorded cortical responses *P*_N1_ and *P*_N5_ between the groups of patients with overt clinical versus subclinical hypothyroidism as well as between the control group versus subclinical hypothyroidism group.

Mann-Whitney's *U* test in study groups			
Potential	Group		
	Control TSH < 3.5	Subclinical 3.5 ≤ TSH ≤ 10	Overt clinical TSH > 10

*P*_N1_ mint		*p = 0.0246*	
	*p* = 0.9903		

*P*_N1_ anise		*p = 0.0114*	
	*p* = 0.6844		

*P*_N5_ mint		*p = 0.0181*	
	*p* = 0.8238		

## References

[1] Misiunas A., Niepomniszcze H., Ravera B., Faraj G., Faure E. (1995). Peripheral neuropathy in subclinical hypothyroidism. *Thyroid*.

[2] Penza P., Lombardi R., Camozzi F., Ciano C., Lauria G. (2009). Painful neuropathy in subclinical hypothyroidism: clinical and neuropathological recovery after hormone replacement therapy. *Neurological Sciences*.

[3] El-Salem K., Ammari F. (2006). Neurophysiological changes in neurologically asymptomatic hypothyroid patients: a prospective cohort study. *Journal of Clinical Neurophysiology*.

[4] Weingaertner J., Proff P., Bienengraeber V., Gedrange T., Fanghaenel J., Lotz K. (2006). In vivo study of apoptosis as a creative agent of embryonic development of the primary nasal duct in rats. *Journal of Cranio-Maxillofacial Surgery*.

[5] Lotz K., Proff P., Bienengraeber V., Fanghaenel J., Gedrange T., Weingaertner J. (2006). Apoptosis as a creative agent of embryonic development of bucca, mentum and nasolacrimal duct. An in vivo study in rats. *Journal of Cranio-Maxillofacial Surgery*.

[6] Mackay-Sim A., Beard M. D. (1987). Hypothyroidism disrupts neural development in the olfactory epithelium of adult mice. *Brain Research*.

[7] Gudziol H., Mlynski G. (1982). Die Olfakto-Rhinorheometrie-eine objektivierende Methode zur Überprüfung des Riechsinnes. *Laryngologie, Rhinologie, Otologie*.

[8] Herberhold C. (1972). Computer-olfaktometrie mit getrenntem nachweis von trigeminus und olfactorius-reaktionen. *Archiv für Klinische und Experimentelle Ohren-, Nasen- und Kehlkopfheilkunde*.

[9] Obrębowski A., Świdziński T., Świdziński P. (2014). Elektrophysiological olfaktometry in clinical practice. *Postępy w Chirurgii Głowy i Szyi*.

[10] McConnell R. J., Menendez C. E., Smith F. R., Henkin R. I., Rivlin R. S. (1975). Defects of taste and smell in patients with hypothyroidism. *The American Journal of Medicine*.

[11] Pruszewicz A., Obrębowski A., Walczak M., Stajgis P., Stawny B., Łącka K. (2004). Przydatność badań węchu i smaku w schorzeniach endokrynologicznych. *Otolaryngologia Polska*.

[12] Walczak M., Pruszewicz A., Łącka K., Karlik M. (2002). Stan narządu powonienia we wrodzonej niedoczynności tarczycy. *Otolaryngologia Polska*.

[13] Walczak M., Pruszewicz A., Łącka K., Karlik M. (2002). Disorders of smell in congenital hypothyroidism. *Polish Journal of Endocrinology*.

[14] Rous J., Synek V. (1966). Objektivni stanoveni intensity cichoveho ujemu pomoci galvanickeho kozniho reflex. *Česka Otolaryngologia*.

[15] Świdziński T., Obrębowski A., Pruszewicz A., Świdziński P. (1999). The use of an automated apparatus for testing olfactory efficiency. *Medical & Biological Engineering & Computing*.

[16] Hummel T., Welge-Lűssen A. (2006). Taste and smell. An update. Assessment of olfactory function. *Advances in Oto-Rhino-Laryngology Home*.

[17] Obrębowski A., Pruszewicz A., Szmeja Z., Rydzewski B., Tyczyńska J. (1977). Olfaktometria obiektywna. *Otolaryngologia Polska*.

[18] Philpott C. M., Wolstenholme C. R., Goodenough P. C., Clark A., Murty G. E. (2006). Comparison of subjective perception with objective measurement of olfaction. *Otolaryngology—Head and Neck Surgery*.

[19] Gudziol H., Gramowski K. H. (1982). Olfacto-EEG-investigations with normal persons. *HNO-Praxis*.

[20] Svitavska A., Uchytil B. (1969). Objektivni olfaktometricky dismulacni test pro overeni anosmickych poruch. *Ceska Otolaryngologia*.

[21] Giesen M., Mrowinski D. (1970). Klinische Untersuchungen mit einem Impuls-Olfactometer. *Sitzungsbericht Freie Vorträge*.

[22] Semeria C. (1956). Studio delle reazioni psico-galvanometrische alla stimolazione olfativa. *Minerva Otorinolaringologica*.

[23] Gerhardt H. J., Rauch Ch. (1963). Objektive Olfaktometrie-Erfahrungen mit Atmungsregistrierung unter Geruchreiz. *Zeitschrift für Laryngologie, Rhinologie, Otologie und ihre Grenzgebiete*.

[24] Roseburg B., Fikentscher R. (1977). *Klinische Olfaktologie und Gustologie*.

[25] Alber K., Mrowiński D., Giesen M., Schwab W. (1972). Objektive Olfaktometrie in der klinischen Diagnostik. *Archiv für Klinische und Experimentelle Ohren-, Nasen- und Kehlkopfheilkunde*.

[26] Fanghänel J., Gedrange T., Proff P. (2006). The face-physiognomic expressiveness and human identity. *Annals of Anatomy*.

[27] Fanghänel J., Gedrange T. (2007). On the development, morphology and function of the temporomandibular joint in the light of the orofacial system. *Annals of Anatomy*.

[28] Kovács T. (2004). Mechanisms of olfactory dysfunction in aging and neurodegenerative disorders. *Ageing Research Reviews*.

[29] Henkin R. I., Levy L. M., Fordyce A. (2013). Taste and smell function in chronic disease: a review of clinical and biochemical evaluations of taste and smell dysfunction in over 5000 patients at the Taste and Smell Clinic in Washington, DC. *American Journal of Otolaryngology—Head and Neck Medicine and Surgery*.

